# Antibiotic-Selected Gene Amplification Heightens Metal Resistance

**DOI:** 10.1128/mBio.02994-20

**Published:** 2021-01-19

**Authors:** David A. Hufnagel, Jacob E. Choby, Samantha Hao, Anders F. Johnson, Eileen M. Burd, Charles Langelier, David S. Weiss

**Affiliations:** a Emory Antibiotic Resistance Center, Atlanta, Georgia, USA; b Emory Vaccine Center, Atlanta, Georgia, USA; c Division of Infectious Diseases, Department of Medicine, Emory University School of Medicine, Atlanta, Georgia, USA; d Division of Infectious Diseases, Department of Medicine, University of California, San Francisco, California, USA; e Department of Microbiology and Immunology, Emory University, Atlanta, Georgia, USA; f Department of Pathology and Laboratory Medicine, Emory University, Atlanta, Georgia, USA; g Chan Zuckerberg Biohub, San Francisco, California, USA; h Research Service, Atlanta VA Medical Center, Decatur, Georgia, USA; University of Texas at Austin; Georgia Institute of Technology School of Biological Sciences

**Keywords:** *Enterobacter*, colistin, gene amplification, heteroresistance, metal resistance, nickel

## Abstract

The increasing frequency of antibiotic resistance poses myriad challenges to modern medicine. Environmental survival of multidrug-resistant bacteria in health care facilities, including hospitals, creates reservoirs for transmission of these difficult to treat pathogens. To prevent bacterial colonization, these facilities deploy an array of infection control measures, including bactericidal metals on surfaces, as well as implanted devices. Although antibiotics are routinely used in these health care environments, it is unknown whether and how antibiotic exposure affects metal resistance. We identified a multidrug-resistant *Enterobacter* clinical isolate that displayed heteroresistance to the antibiotic colistin, where only a minor fraction of cells within the population resist the drug. When this isolate was grown in the presence of colistin, a 9-kb DNA region was duplicated in the surviving resistant subpopulation, but surprisingly, was not required for colistin heteroresistance. Instead, the amplified region included a three-gene locus (*ncrABC*) that conferred resistance to the bactericidal metal, nickel. *ncrABC* expression alone was sufficient to confer nickel resistance to E. coli K-12. Due to its selection for the colistin-resistant subpopulation harboring the duplicated 9-kb region that includes *ncrABC*, colistin treatment led to enhanced nickel resistance. Taken together, these data suggest that the use of antibiotics may inadvertently promote enhanced resistance to antimicrobial metals, with potentially profound implications for bacterial colonization and transmission in the health care environment.

## OBSERVATION

Increasing resistance to antibiotics is a threat to modern medicine, in some cases precluding the treatment of bacterial infections and routine procedures that rely on these drugs such as surgeries, cancer chemotherapy, and organ transplantations. One prediction suggests that 10 million people will die worldwide each year from antibiotic-resistant infections by 2050 (1). In the United States alone, there are currently estimated to be over 150,000 annual deaths due to antibiotic-resistant bacteria (2). Infections caused by the carbapenem-resistant *Enterobacterales* (CRE; including *Enterobacter*, *Escherichia*, and *Klebsiella*) are one of the most worrisome threats since they increasingly require treatment with last-line antibiotics such as colistin and in certain cases are resistant to all available drugs (3).

*Enterobacterales* can persist in the environment for months, including in hospitals, which increases the opportunities for transmission of these pathogens to new hosts (4, 5). In fact, there are more than 700,000 health care associated infections each year in the United States, many of which are likely linked to the ability of bacteria to persist in the environment (6). Astoundingly, one study found that 92% of patients with a CRE infection had in the past year visited a health care facility (75.1% had acute care hospitalization), where rampant antibiotic use routinely selects for highly resistant bacteria (6, 7). To curb transmission of such pathogens to hospitalized patients, antimicrobial metals are utilized on patient contact surfaces and medical devices (8, 9). While other studies have demonstrated that metal exposure can induce antibiotic resistance, it is unclear whether antibiotics affect bacterial metal resistance and thus capacity to colonize antimicrobial metal surfaces.

**FIG 1:**
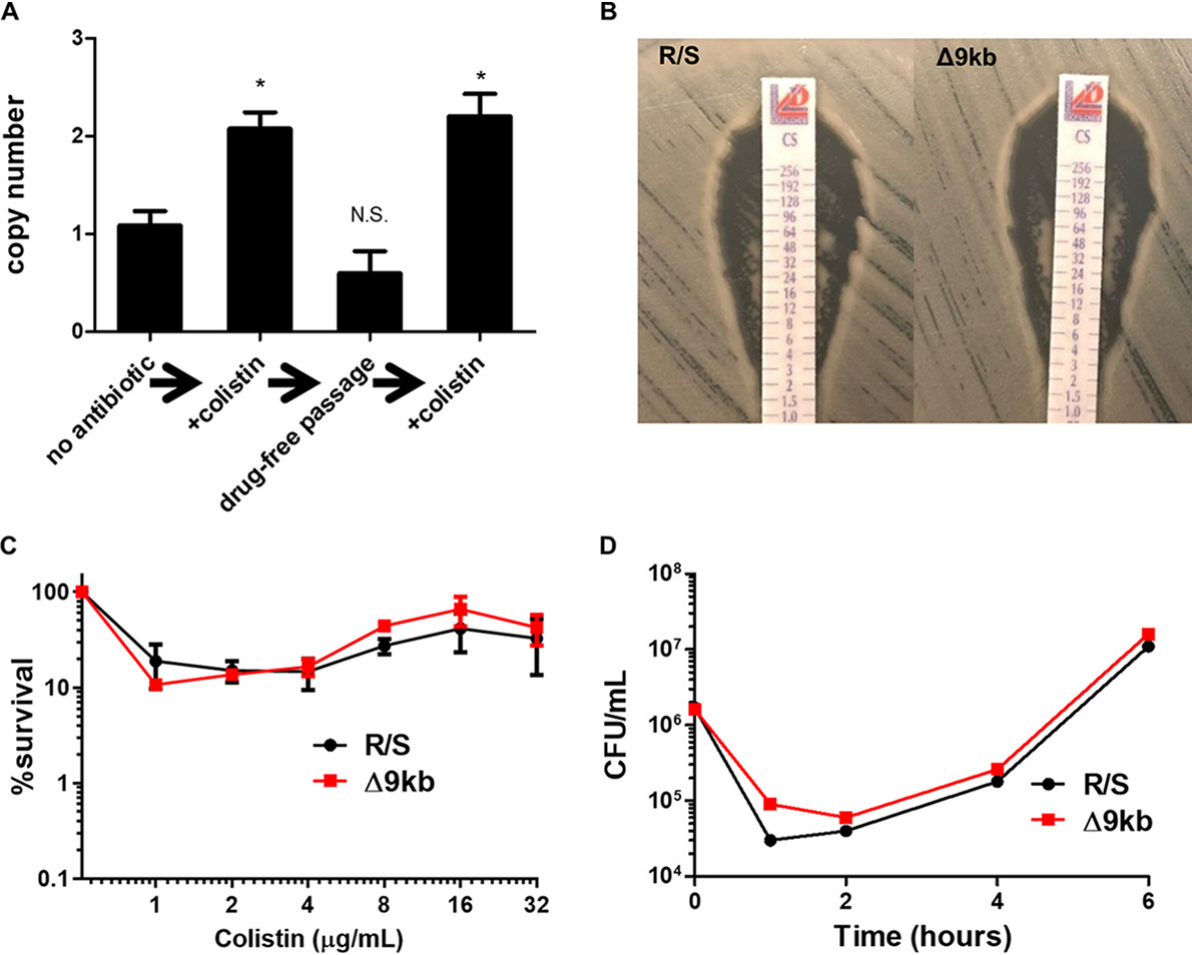
DNA duplication selected by colistin does not confer colistin resistance. (A) Quantitative PCR for a 150-bp segment within the 9-kb region, as well as the housekeeping control gene *rpoD*, was performed on R/S genomic DNA after sequential subculture and growth in Mueller-Hinton medium (MH; no antibiotic), MH + 100 µg/ml colistin (+colistin), drug-free MH medium (drug-free passage), and MH + 100 µg/ml colistin (+colistin). (B to D) Comparison of R/S and a deletion mutant lacking the 9-kb region (Δ9kb) by colistin gradient test strip (B), population analysis profile on MH plates with the indicated concentrations of colistin (C), and time-kill analysis over 6 h in colistin (100 µg/ml) (D). Significance values determined by using a Student two-tailed *t* test (*, *P* < 0.05). For panel A, the means of three biological replicates performed with technical duplicates and standard errors of the mean are shown. For panel C, the means and standard deviations of a representative experiment with biological duplicates are shown. For panel D, a representative experiment is shown with one replicate per strain.

In studying human clinical isolates of Enterobacter cloacae from the health care setting, we identified a strain (“R/S”) that exhibits heteroresistance to colistin (10, 11). Heteroresistance is a phenomenon where a minor group of cells in a population are resistant to an antibiotic and coexist with a majority susceptible population (12). The growth of R/S in colistin (100 µg/ml in Mueller-Hinton medium [MH]) selected for the resistant subpopulation; however, subsequent drug-free passage resulted in a return to the baseline frequency of 10% resistant cells (see Fig. S1A in the supplemental material) (11). Analysis of mapped reads from Illumina whole-genome sequencing of R/S grown in the presence or absence of colistin revealed identical sequences with no genetic differences (11). However, further analysis of gene copy number revealed a single 9-kb region that was duplicated exclusively when R/S was grown in the presence of colistin (see Fig. S1B). Interestingly, this 9-kb region is flanked by transposon-related elements, suggesting gene amplification might rely on these sequences, as has been observed for antibiotic resistance genes and a nickel resistance locus in *Pseudomonas* (13, 14). Using quantitative PCR (qPCR) on genomic DNA isolated from R/S grown with or without colistin, we confirmed the duplication of the 9-kb region in colistin-treated cultures (Fig. 1A). Subsequent overnight passage in the absence of colistin resulted in reversion to a single copy of the 9-kb region and reexposure of the passaged strain to colistin led to its reamplification (Fig. 1A), correlating with the colistin resistance dynamics displayed by R/S (see Fig. S1A) (11). In contrast, when R/S was challenged with 0.25× MIC of other antibiotics (amikacin, cefepime, or ciprofloxacin), we observed no duplication of the 9-kb region, highlighting the specificity of this selection (see Fig. S1C).

10.1128/mBio.02994-20.1 FIG S1 Selection for a 9-kb DNA amplification occurs in the presence of colistin. (A) R/S was sequentially subcultured and grown in Mueller-Hinton (MH) medium (no antibiotic), MH + 100 µg/ml colistin (+colistin), drug-free MH medium (drug free passage), and MH + 100 µg/ml colistin (+colistin) and at each step plated on MH plates with or without colistin (100 µg/ml) to determine the percent survival. (B) Comparison of raw unfiltered reads from whole-genome sequencing of biological triplicates of R/S grown with or without colistin 100 µg/ml. The 9-kb region was the only amplification identified. (C) Genomic DNA was collected from R/S grown in MH with or without colistin (100 µg/ml), amikacin (0.5 µg/ml), cefepime (2 µg/ml), or ciprofloxacin (8 µg/ml). Quantitative PCR was used to compare the copy number of the 9-kb region relative to the chromosomal gene *rpoD.* (D) R/S, Δ9kb, and Δ*ncrABC* strains were grown at 37°C with shaking at 250 rpm in Mueller-Hinton (MH) medium. The CFU/ml were enumerated after plating at the indicated time points. Significance values determined by using a Student two tailed *t* test (*, *P* < 0.05; **, *P* < 0.01). For panel A, the results of a representative experiment are shown with the means and standard deviations from three biological replicates. For panel C, the means of three biological replicates performed with technical duplicates and standard errors of the mean are shown. For panel D, the results of a representative experiment are shown with the means and standard deviations of two biological replicates. Download FIG S1, TIF file, 0.6 MB. Copyright © 2021 Hufnagel et al. 2021 Hufnagel et al. This content is distributed under the terms of the Creative Commons Attribution 4.0 International license.

To investigate the potential role of the 9-kb region in colistin heteroresistance, we used lambda red mutagenesis (15, 16) to generate an R/S isogenic mutant completely lacking the 9-kb region (Δ9kb). Surprisingly, R/S and Δ9kb had a similar frequency of resistant colonies growing in the zone of inhibition when plated with a colistin MIC test gradient strip (0.5 McFarland was used for spread plating [Liofilchem, Waltham, MA]), indicating the 9-kb region was not required for colistin heteroresistance (Fig. 1B). Similarly, population analysis profile, which is used to quantify bacterial subpopulations via serial dilution and plating on doubling dilutions of antibiotic to determine the percent survival (12), revealed no difference in the frequency of the colistin-resistant subpopulation between R/S and Δ9kb (Fig. 1C). Finally, treatment of R/S and Δ9kb with colistin in broth indicated that each strain harbored a resistant subpopulation that was able to rapidly expand in the antibiotic (Fig. 1D). Taken together, these data show that despite the amplification of the 9-kb region during colistin exposure, the corresponding genes do not contribute to colistin heteroresistance.

**FIG 2:**
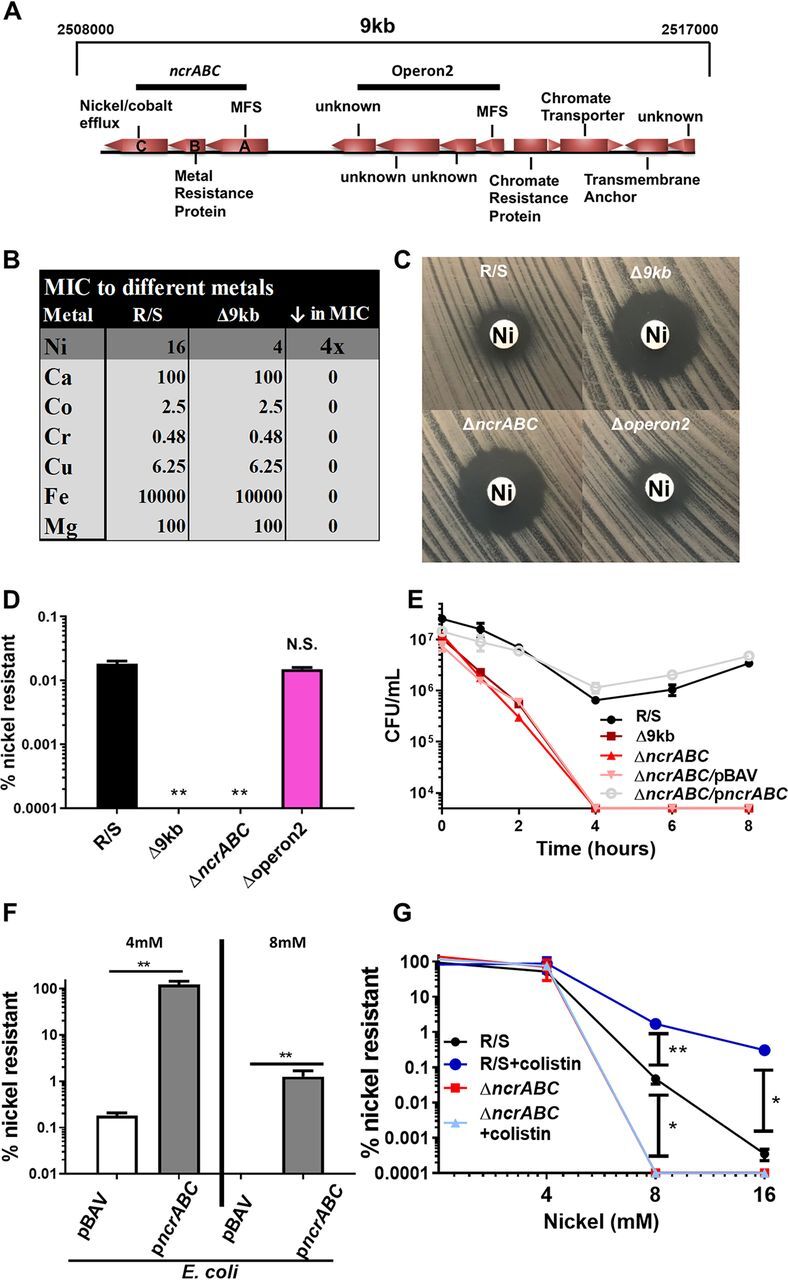
Colistin-selected DNA duplication confers resistance to nickel. (A) Schematic of a 9-kb region duplicated in Enterobacter cloacae strain R/S upon growth in colistin as detected by genome sequencing and mapping of raw, unfiltered reads. MFS, major facilitator superfamily. (B) Broth microdilution of various metals on R/S and Δ9kb to determine MICs [Ni, nickel(II) sulfate; Ca, calcium chloride; Co, cobalt chloride; Cr, sodium chromate; Cu, copper(II) sulfate; Fe, iron(II) sulfate; Mg, magnesium chloride]. (C) Discs containing 1.5 mg of nickel(II) sulfate were added to 0.5 McFarland standard of R/S and strains lacking 9-kb, *ncrABC*, and operon2 spread plates. (D) R/S and isogenic mutants were plated with or without 8 mM nickel(II) sulfate to determine the percent survival of each isolate. (E) 10^7^ CFU of R/S and the indicated mutants were grown in 8 mM nickel(II) sulfate, and the surviving CFU were determined at the indicated time points. (F) E. coli K-12 BW25113/pBAV and p*ncrABC* were plated with or without 4 or 8 mM nickel(II) sulfate to determine the percent survival of each isolate. (G) Nickel population analysis profile of R/S and Δ*ncrABC* strains grown for 5 h in MH with or without 100 µg/ml colistin prior to CFU enumeration of plates containing various concentrations of nickel(II) sulfate. Significance values determined by using a Student two-tailed *t* test (*, *P* < 0.05; **, *P* < 0.01). For panels D, E, and G, the data shown are as means with the standard deviations for a single representative experiment with three biological replicates. For panel F, the data shown as means and standard deviations of two independent experiments, each with biological replicates.

Since the 9-kb region did not affect colistin resistance, we next determined its physiological role. While there were no known colistin resistance genes present within this region, bioinformatic analysis revealed that it contained multiple putative metal resistance genes (Fig. 2A). We therefore quantified the MIC of R/S and Δ9kb to a panel of metals via broth microdilution (BMD) (Fig. 2B). Briefly, 5 × 10^5^ CFU of bacteria were inoculated into a 96-well plate with doubling dilutions of metals and incubated for 20 h at 37°C. Of the metals tested, the 9-kb mutation only reduced the MIC of R/S to nickel [nickel(II) sulfate; Alfa Aesar, Ward Hill, MA] (Fig. 2B). Disk diffusion (6-mm paper discs; BBL, Sparks, MD) similarly indicated an increased susceptibility of Δ9kb to nickel compared to R/S (Fig. 2C).

To identify genes within the 9-kb region that confer enhanced resistance to nickel, we mutated two putative metal resistance operons containing major facilitator superfamily (MFS) genes (Fig. 2A). Disk diffusion analysis revealed that one of the putative operon mutants phenocopied the increased nickel susceptibility of Δ9kb, and the deleted genes were termed *ncrABC* based on high sequence similarity (100% amino acid and 99% nucleotide) to nickel resistance genes first described in Leptospirillum ferriphilum (17) (Fig. 2C). The second mutant lacking a putative operon (Δoperon2) displayed a similar level of nickel resistance as R/S (Fig. 2C). In addition, the Δ9kb and Δ*ncrABC* strains had decreased survival on nickel agar plates compared to R/S, whereas Δoperon2 phenocopied R/S (Fig. 2D). Both Δ9kb and Δ*ncrABC* strains had similar growth kinetics as R/S in MH, highlighting that the nickel susceptibility of these strains was not due to an underlying fitness defect (see Fig. S1D). In addition, the *ncrABC* mutation did not affect the resistance of R/S to a panel of antibiotics as tested via Vitek 2 (bioMérieux, Marcy l’Étoile, France), indicating that the contribution of *ncrABC* is specific (see Table S1 in the supplemental material). Importantly, in *trans* complementation of the Δ*ncrABC* (Δ*ncrABC*/p*ncrABC*) strain restored survival in the presence of nickel, while the Δ*ncrABC* and Δ9kb strains were killed (Fig. 2E). Further, the Δ*ncrABC*/p*ncrABC* strain exhibited a restored nickel MIC of 16 mM by BMD, similar to R/S and compared to an MIC of 4 mM for the Δ9kb and Δ*ncrABC* strains (see Fig. S2A). These data suggest the *ncrABC* genes are important for nickel resistance in R/S, despite the presence of a homolog of the RcnA nickel resistance protein (59% amino acid identity to E. coli K-12 RcnA) encoded elsewhere in the genome. The Δ*ncrABC*/p*ncrABC* strain also exhibited increased survival by nickel disk diffusion and had no effect on fitness in MH (see Fig. S2B and C). Disk diffusion on minimal media similarly revealed that *ncrABC* deletion most robustly decreased nickel resistance (see Fig. S2D). In contrast, *ncrABC* deletion had no effect on chromium, copper, or iron resistance, but interestingly, cobalt resistance was slightly reduced in the Δ*ncrABC* mutant (see Fig. S2D). These data clearly indicate that *ncrABC* mediate nickel resistance in R/S.

10.1128/mBio.02994-20.2 FIG S2 *ncrABC* genes are sufficient for nickel resistance. (A) Nickel(II) sulfate MICs were determined for R/S, Δ9kb, Δ*ncrABC*, and Δ*ncrABC/*p*ncrABC* strains by broth microdilution (BMD) with biological replicates. 5 × 10^5^ CFU were inoculated into 100 µl of Mueller-Hinton (MH) broth containing doubling dilutions of nickel. The lowest concentration of nickel that inhibited turbidity at 20 h at 37°C was determined as the MIC. (B) 0.5 McFarland Δ*ncrABC/*pBAV and Δ*ncrABC/*p*ncrABC* were spread onto MH plates. Discs (6 mm) containing 1.5 mg of nickel(II) sulfate were added prior to incubation at 37°C for 20 h. (C) Δ*ncrABC/*pBAV and Δ*ncrABC/*p*ncrABC* strains were grown in MH medium at 37°C and 250 rpm. The CFU were enumerated at the indicated time points. (D) 0.5 McFarland R/S and Δ*ncrABC* M9-dextrose (0.5%)-grown cells were spread plated onto M9-dextrose plates. Then, 20 µl of metal solutions of Ni (500 mM), Co (5 mM), Cr (500 mM), Cu (1 M), or Fe (1 M) were added to paper discs on the spread bacteria; the plates were incubated for 37°C for 20 h, and the diameters of inhibition were determined. The fold change is the diameter of Δ*ncrABC* divided by the diameter of R/S to the same metal. For panel C, the results of a representative experiment are shown with the means and standard deviations of three biological replicates shown. For panel D, the results of a representative experiment are shown with the means and standard deviations of two biological replicates. Download FIG S2, TIF file, 0.5 MB. Copyright © 2021 Hufnagel et al. 2021 Hufnagel et al. This content is distributed under the terms of the Creative Commons Attribution 4.0 International license.

10.1128/mBio.02994-20.9 TABLE S1 *ncrABC* mutation does not affect resistance to tested antibiotics. R/S and Δ*ncrABC* MICs (µg/ml) were determined to a panel of antibiotics via Vitek 2 or gradient test strip (colistin) (bioMerieux). SXT, trimethoprim sulfamethoxazole; Pip/Tazo, piperacillin-tazobactam. Download Table S1, DOCX file, 0.01 MB. Copyright © 2021 Hufnagel et al. 2021 Hufnagel et al. This content is distributed under the terms of the Creative Commons Attribution 4.0 International license.

To determine whether *ncrABC* were sufficient for nickel resistance, we expressed these genes in E. coli K-12 and observed a >1,000-fold increase in survival in the presence of nickel (Fig. 2F). In addition, upon expression of *ncrABC*, the BMD nickel MIC of E. coli increased from 4 mM to 16 mM, the level observed for R/S (see Fig. S3). These data indicate that the *ncrABC* genes are sufficient to confer nickel resistance.

10.1128/mBio.02994-20.3 FIG S3 *ncrABC* is sufficient for increasing nickel MIC. E. coli*/*pBAV and E. coli*/*p*ncrABC* MICs to nickel(II) sulfate were determined by broth microdilution (BMD) in biological triplicate after 20 h incubation at 37°C. Download FIG S3, TIF file, 0.1 MB. Copyright © 2021 Hufnagel et al. 2021 Hufnagel et al. This content is distributed under the terms of the Creative Commons Attribution 4.0 International license.

Since the *ncrABC* mutation confers nickel resistance and is encoded within a region of DNA that was duplicated upon colistin treatment, we hypothesized that colistin would lead to enhanced nickel resistance due to increased *ncrABC* gene dosage. Indeed, we observed an *ncrABC*-dependent increase in nickel resistance when R/S was grown with colistin (Fig. 2G). Importantly, genes in the 9-kb region still duplicated in the Δ*ncrABC* mutant in the presence of colistin, indicating that the lack of colistin-dependent nickel resistance in the Δ*ncrABC* strain was not due to an abrogation of amplification of this region (see Fig. S4). To determine whether a cationic molecule other than colistin would induce nickel resistance, we grew R/S in minimal media with or without iron(II) sulfate (+Fe) and assayed for both survival in nickel and the nick zone of inhibition by disk diffusion assay (see Fig. S5A and B). While colistin increased nickel resistance in both assays, iron did not affect nickel resistance in R/S (see Fig. S5A and B).

10.1128/mBio.02994-20.4 FIG S4 Colistin-selected locus amplification does not require *ncrABC. ΔncrABC* was grown with or without 100 µg/ml colistin for 4 h at 37°C prior to genomic DNA isolation. Quantitative PCR was used to determine the relative copies of the 9-kb region without *ncrABC*. Significance values determined by using a Student two-tailed *t* test (*, *P* < 0.01). The means of four biological replicates performed with technical duplicates and standard errors of the mean are shown. Download FIG S4, TIF file, 0.06 MB. Copyright © 2021 Hufnagel et al. 2021 Hufnagel et al. This content is distributed under the terms of the Creative Commons Attribution 4.0 International license.

10.1128/mBio.02994-20.5 FIG S5 Iron supplementation does not induce enhanced nickel resistance in R/S. (A) 0.5 McFarland R/S grown in M9-dextrose (0.5%) supplemented with or without 5 mM Fe or 100 µg/ml colistin were plated onto minimal media plates with or without 24 mM nickel(II) sulfate. The percent survival was determined by dividing the CFU growing with nickel versus nickel-free plates. (B) 0.5 McFarland R/S grown in M9-dextrose with or without 5 mM Fe or 100 µg/ml colistin were spread plated onto M9-dextrose plates. Discs containing 20 µl of 500 mM nickel were added to the lawn of bacteria, and plates were incubated at 37°C for 20 h before the diameters of the zones of inhibition were determined. The fold change in the diameter of the zone of inhibition was determined by dividing the zone of inhibition under each condition by the average zone of inhibition of R/S grown in M9-dextrose without supplementation (M9). Significance values determined by using a Student two-tailed test (*, *P* < 0.01). For panel A, the results of a representative experiment are shown with the means and standard deviations of two biological replicates. For panel B, the results of a representative experiment are shown with the means and standard deviations of three biological replicates. Download FIG S5, TIF file, 0.1 MB. Copyright © 2021 Hufnagel et al. 2021 Hufnagel et al. This content is distributed under the terms of the Creative Commons Attribution 4.0 International license.

We next determined whether colistin-induced nickel resistance was widespread or an isolated phenotype. A nucleotide alignment of the entire 9-kb region revealed it is present in over 83 sequenced bacterial genomes with >80% nucleotide identity and >70% query coverage, all of which are gammaproteobacteria. With the exception of 1 *Shewanella* and 2 *Serratia* genomes, the rest are *Enterobacterales*: 33 *Klebsiella*, 29 *Enterobacter*, 11 *Citrobacter*, 3 *Escherichia*, 2 *Raoultella*, a *Phytobacter*, and 1 *Metakosakonia*. Based on these findings, with our stringent alignment parameters, the 9-kb region may not be widely present in all isolates of a particular *Enterobacterales* species but is widely present in many species. As an example, Enterobacter cloacae clinical isolate Mu208 was observed to encode the same 9-kb region as R/S. We found that the 9-kb region in Mu208 also duplicated in the presence of colistin (see Fig. S6A in the supplemental material), contributed to survival on nickel (see Fig. S6B), and increased *ncrABC-*dependent nickel resistance when grown in the presence of colistin (see Fig. S6B). These data suggest that colistin-induced, *ncrABC*-dependent nickel resistance is likely a broadly relevant phenomenon.

10.1128/mBio.02994-20.6 FIG S6 Mu208 exhibits *ncrABC-*dependent nickel resistance. (A) The Mu208 9-kb region duplicates in response to colistin exposure. DNA was isolated from Mu208 grown in MH or MH with colistin (100 µg/ml) and subjected to qPCR for a genomic control gene (*rpoD*) and the 9-kb region (using *ncrC* as a marker). The fold change was calculated to determine the copy number. (B) *ncrABC*-dependent nickel resistance is heightened by colistin exposure. Mu208 WT and Δ*ncrABC* stains were grown in MH with or without colistin (100µg/ml), and the percent survival was determined on 6 mM nickel agar plates. Significance values were determined by using a Student two-tailed *t* test (**, *P* < 0.01). For panel A, the means of three biological replicates performed with technical duplicates and the standard errors of the mean are shown. For panel B, the means and standard deviations are shown from a representative experiment with three biological replicates. Download FIG S6, TIF file, 0.1 MB. Copyright © 2021 Hufnagel et al. 2021 Hufnagel et al. This content is distributed under the terms of the Creative Commons Attribution 4.0 International license.

In addition, further bioinformatic analyses allowed us to make insights into the regulation of the *ncrABC* operon. NcrA is a predicted MFS family protein (Pfam PF07690) and NcrC is a predicted transmembrane protein which has amino acid analogy to the Ni and Co efflux proteins NirC (Pfam PF03824) (17, 18) and RcnA (19) in *Enterobacterales.* NcrB is a predicted helix-turn-helix transcriptional regulator with structural homology to RcnR and CsoR, which are part of a family of metal-sensing negative regulators of metal efflux systems in other organisms (Pfam PF02583) (20). NcrB contains 9 histidine residues (out of 87 amino acids), and this amino acid is known to bind nickel, suggesting that NcrB could be a regulator of *ncrABC*. We first showed that growth of wild-type (WT) R/S in the presence of nickel resulted in a marked increase in the expression level of *ncrC* (see Fig. S7A). Consistent with NcrB functioning as a negative regulator of the operon, deletion of *ncrB* led to a significant increase in expression of *ncrC* (see Fig. S7B). In addition to the expression data, the *ncrB* deletion strain exhibited a robust increase in nickel resistance while *ncrA* or *ncrC* mutants had a decrease in nickel resistance (see Fig. S7C). It is interesting that the *ncrA* and *ncrC* mutants have modest but complementable phenotypes (see Fig. S7D and E) relative to the *ncrABC* deletion strain, suggesting that they may have some overlapping function that is apparent only when both are deleted. The enhanced nickel resistance of Δ*ncrB* correlated with decreased intracellular levels of this metal relative to WT R/S as measured by ICP-AES (Thermo iCAP 7400) (see Fig. S7F). Taken together, these data demonstrate that colistin selects for a nickel-resistant subpopulation of R/S cells harboring an amplification of a nickel resistance locus and highlight that antibiotic treatment can promote increased metal resistance (see Fig. S8).

10.1128/mBio.02994-20.7 FIG S7 *ncrABC* expression is governed by nickel and NcrB, and each gene affects nickel resistance differently. (A) RNA was harvested from R/S grown 4 h in MH with or without 8 mM nickel as indicated. qRT-PCR analysis was performed on RNA with primers for *ncrC* or *rpoD* (housekeeping gene). Expression levels were determined by 2^–ΔΔ^*^CT^*. (B) RNA was harvested from R/S and Δ*ncrB* grown 4 h in MH. qRT-PCR analysis was performed on RNA with primers for *ncrC* or *rpoD* (housekeeping gene). Expression levels were determined by 2^–ΔΔ^*^CT^*. (C) Lambda red mutagenesis constructed mutants *ncrABC*, *ncrA*, *ncrB*, and *ncrC* were serially diluted and plated on MH plates supplemented with increasing doubling dilutions of nickel. (D and E) R/S and respective mutants were grown with empty vector (pBAV) or the indicated complementation vector overnight in Mueller-Hinton broth containing 35 µg/ml kanamycin for plasmid selection, serially diluted, and plated on MH plates supplemented with 8 mM (D) or 12 mM (E) nickel. The percent survival was determined by the ratio of CFU in the presence of nickel divided by the CFU on unsupplemented plates × 100. (F) R/S and Δ*ncrB* strains were grown for 4.5 h under *ncrABC*-inducing conditions (Mueller-Hinton broth supplemented with 1 mM nickel). Then, 13 ml of 0.5 OD_600_ cells were pelleted and washed three times in metal-free H_2_O. The pellets were digested with nitric acid and H_2_O_2_ prior to analysis by ICP-AES (Thermo iCAP 7400 [LIME Penn State University]) to determine nickel concentrations. For panels A and B, the means of three biological replicates performed with technical duplicates and the standard errors of the mean are shown. Significance values determined by using a Student two-tailed *t* test (**, *P* < 0.01). For panel C, a representative experiment with one replicate of each strain is shown. For panels D and E, the significance values were determined by using a Kruskal-Wallis test comparing each to R/S pBAV in panel D and the comparisons indicated in panel E; the data show the means and standard deviations of data from two independent experiments with at least three biological replicates each (*, *P* < 0.05; ***, *P* < 0.001). For panel F, significance values were determined by using a Student two-tailed *t* test (**, *P* < 0.01). The means and standard errors of the mean are shown from a representative experiment with three biological replicates. Download FIG S7, TIF file, 0.4 MB. Copyright © 2021 Hufnagel et al. 2021 Hufnagel et al. This content is distributed under the terms of the Creative Commons Attribution 4.0 International license.

10.1128/mBio.02994-20.8 FIG S8 Potential effects of antibiotic-selected metal-resistant subpopulations in a hospital environment. The schematic shows an infected patient who is or is not treated with colistin. In the absence of colistin, cells harboring the duplication of *ncrABC* are not selected; the bacteria are susceptible to nickel and are unable to colonize a metal-coated surface in the hospital. In contrast, colistin treatment selects for cells with two copies of *ncrABC*, which facilitates increased resistance to the bactericidal metal nickel, which could lead to enhanced environmental survival in the healthcare environment. Download FIG S8, TIF file, 0.3 MB. Copyright © 2021 Hufnagel et al. 2021 Hufnagel et al. This content is distributed under the terms of the Creative Commons Attribution 4.0 International license.

Amplification of antibiotic resistance genes has recently been demonstrated to occur in some examples of heteroresistance (21, 22). This leads to a subpopulation of cells with higher antibiotic resistance gene dosage and increased resistance. In the present study of a colistin-heteroresistant isolate, we observe a gene amplification that is selected by the antibiotic (colistin) but which is not a determinant of the heteroresistance phenotype. Therefore, it is important to note that the observation of a gene amplification upon treatment with a specific stress (i.e., antibiotic) should not automatically be interpreted as indicating that the amplified gene(s) mediate the heteroresistance phenotype.

Multiple studies have found that bacterial exposure to metals can confer resistance to antibiotics and that metal and antibiotic resistance genes are often encoded on the same mobile genetic elements (23–26). In contrast, the effect of antibiotics on bacterial metal resistance has been unclear. The present study shows that antibiotic treatment can lead to metal resistance since colistin led to enhanced nickel resistance via duplication of *ncrABC*. These findings highlight an important consequence of antibiotic use, warning that these drugs could prime bacterial populations for survival on bactericidal metal-coated surfaces and thus enhance colonization of the hospital environment, leading to subsequent transmission and infection (see Fig. S8).

### Data availability.

All data are provided in the manuscript, in the supplemental materials, or are available upon request from the authors. The primer and nucleotide list (see Table S2 in the supplemental material) includes sequences and cloning information referenced in the manuscript. The DNA sequencing data have been deposited at NCBI under BioProject no. PRJNA263343.

10.1128/mBio.02994-20.10 TABLE S2 Primer and nucleotide list. This list contains primer names and sequences that were utilized throughout the manuscript, along with the 9-kb region nucleotide sequence. Download Table S2, DOCX file, 0.02 MB. Copyright © 2021 Hufnagel et al. 2021 Hufnagel et al. This content is distributed under the terms of the Creative Commons Attribution 4.0 International license.
