# Supplementary figures and images for: Antibiotic-Selected Gene Amplification Heightens Metal Resistance
Source: mBio. 2021 Jan 19;12(1):e02994-20. doi: 10.1128/mBio.02994-20 (PMC8545094; doi:10.1128/mBio.02994-20)

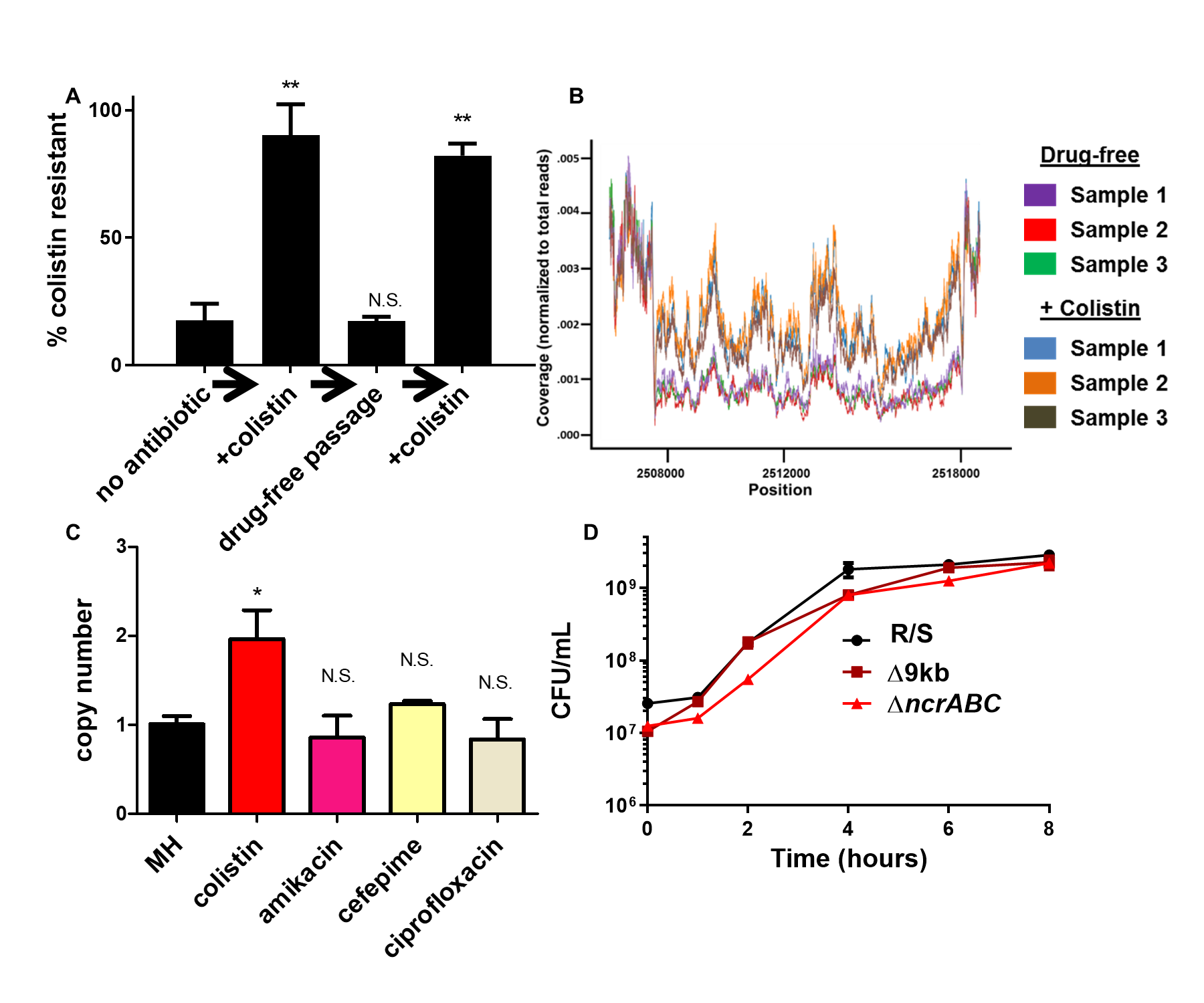

Supplement: FIG S1 [file mbio.02994-20-sf001.tif]

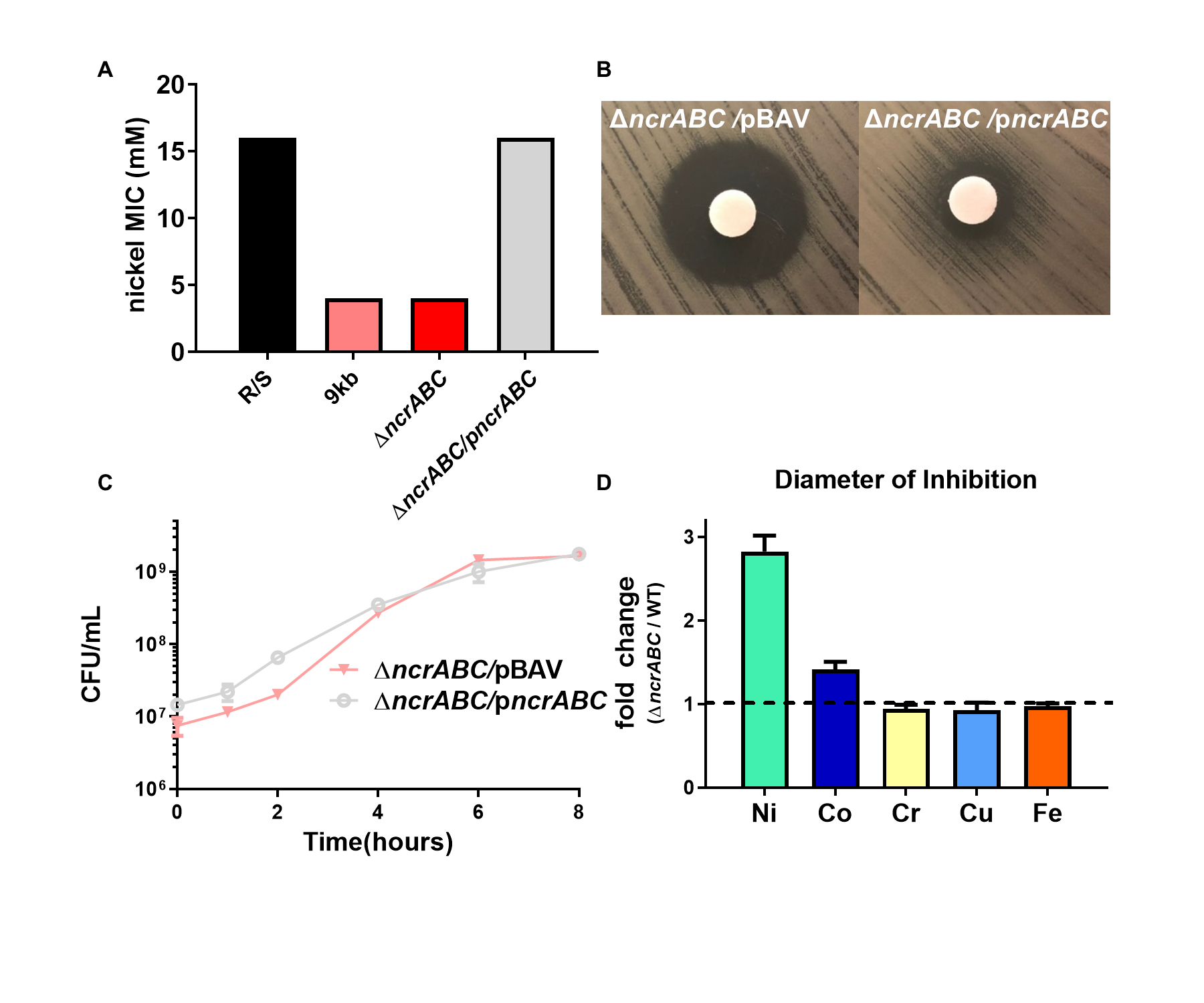

Supplement: FIG S2 [file mbio.02994-20-sf002.tif]

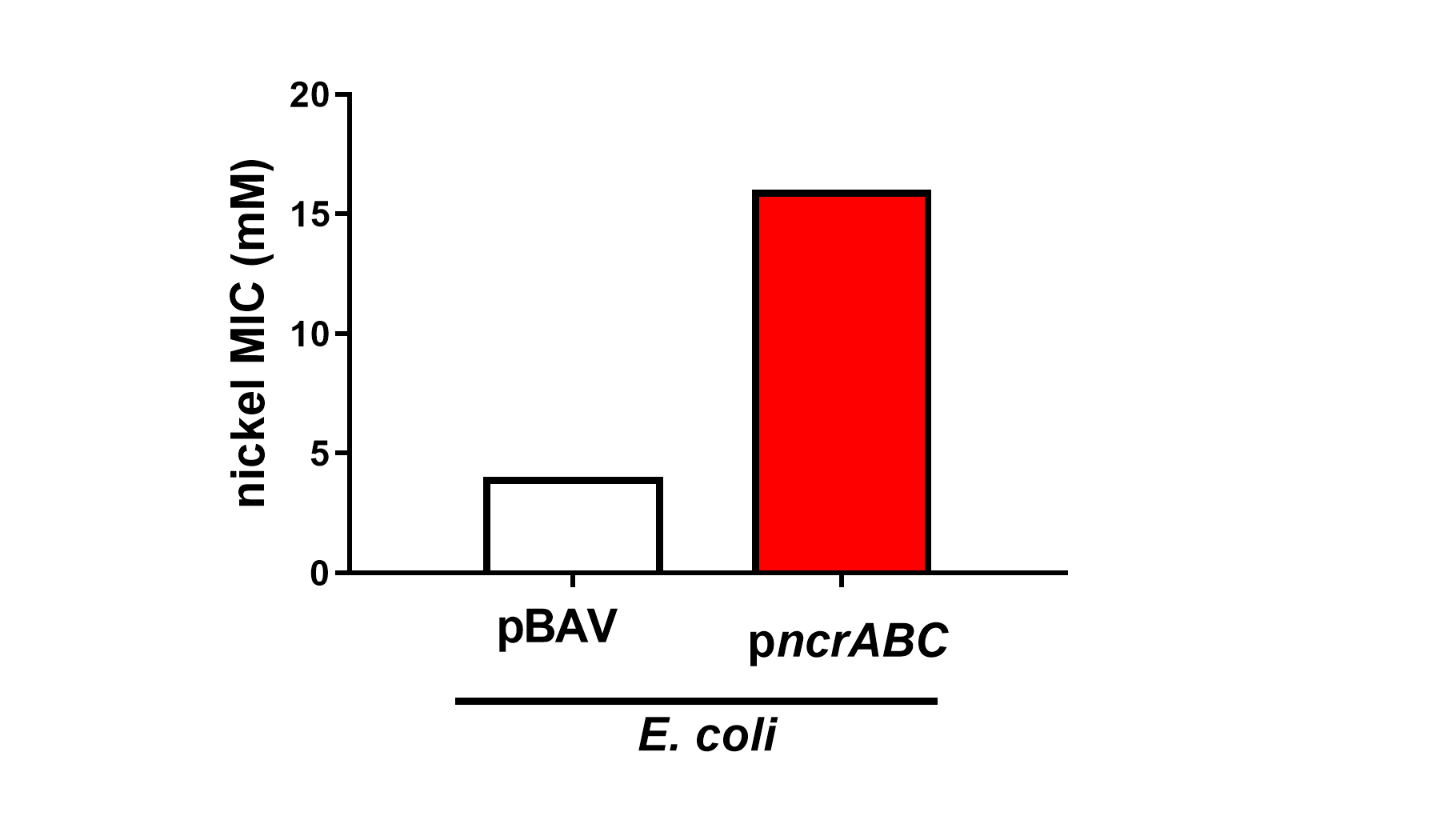

Supplement: FIG S3 [file mbio.02994-20-sf003.tif]

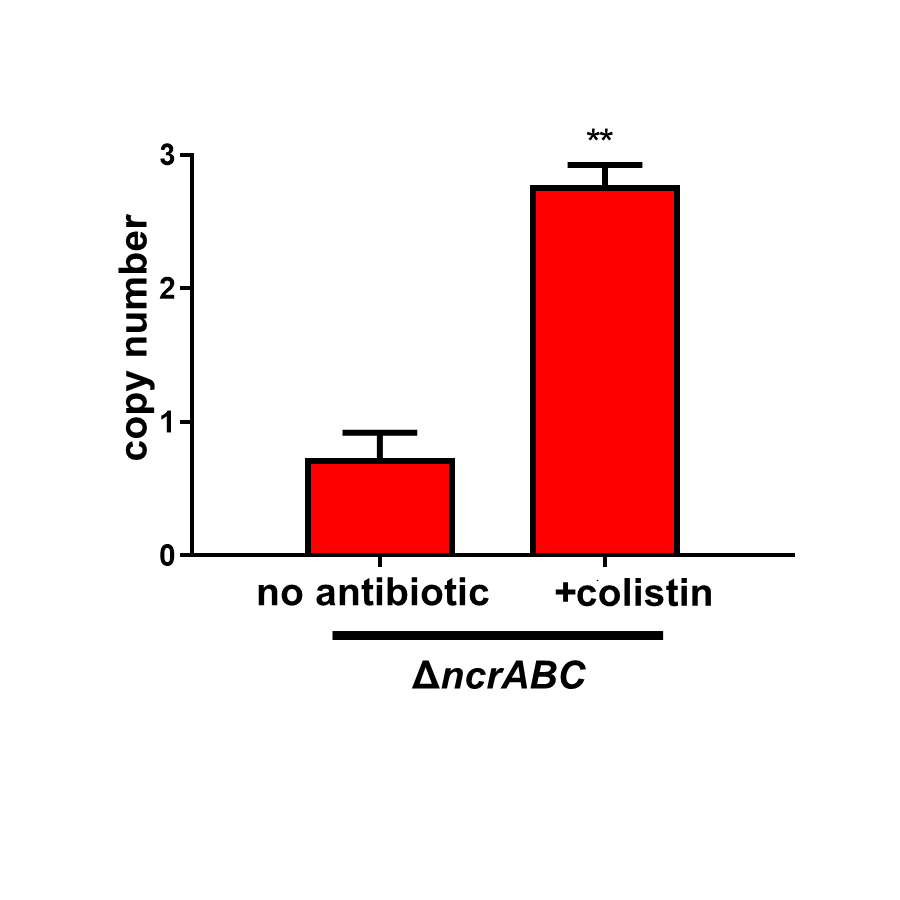

Supplement: FIG S4 [file mbio.02994-20-sf004.tif]

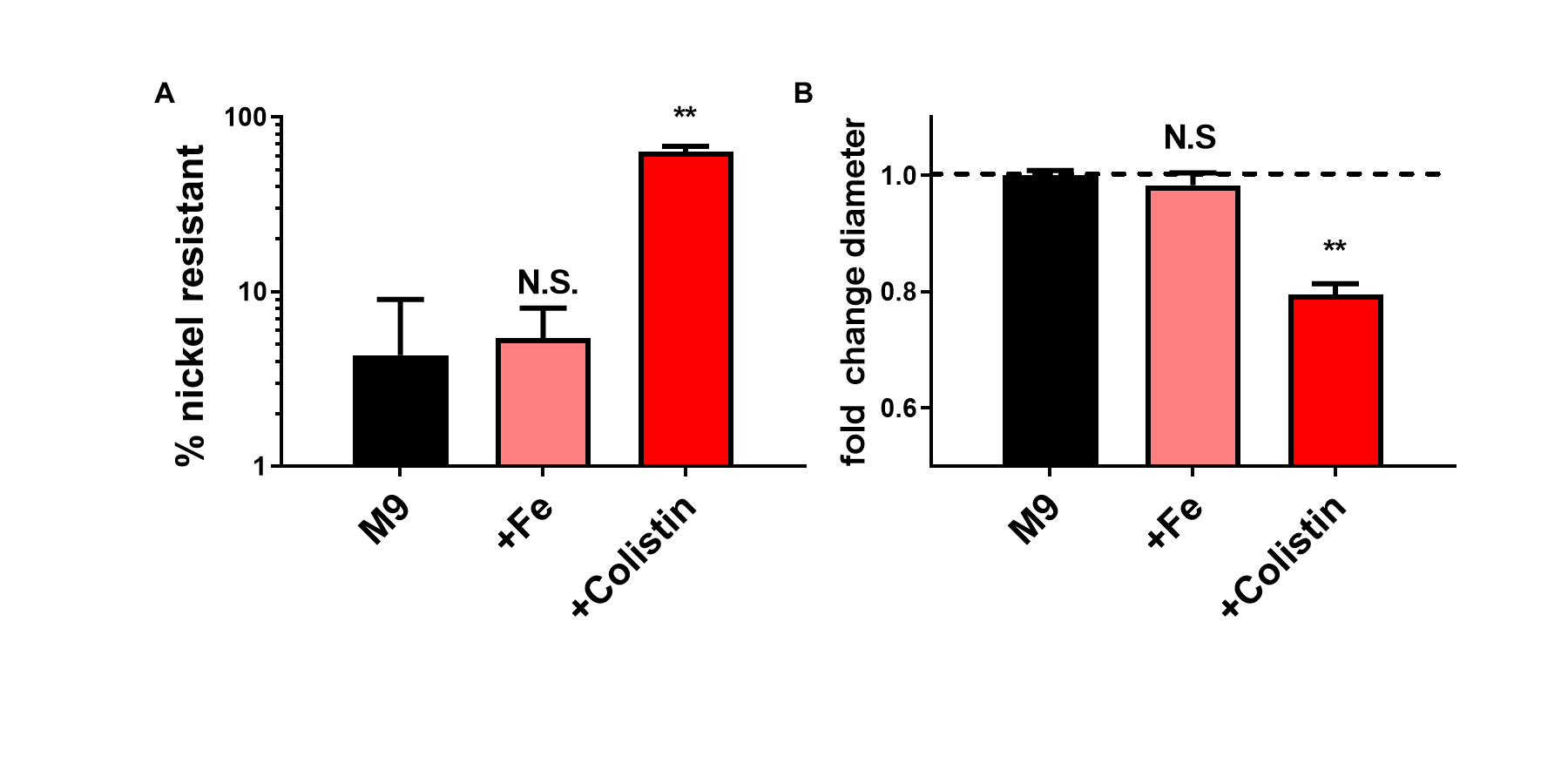

Supplement: FIG S5 [file mbio.02994-20-sf005.tif]

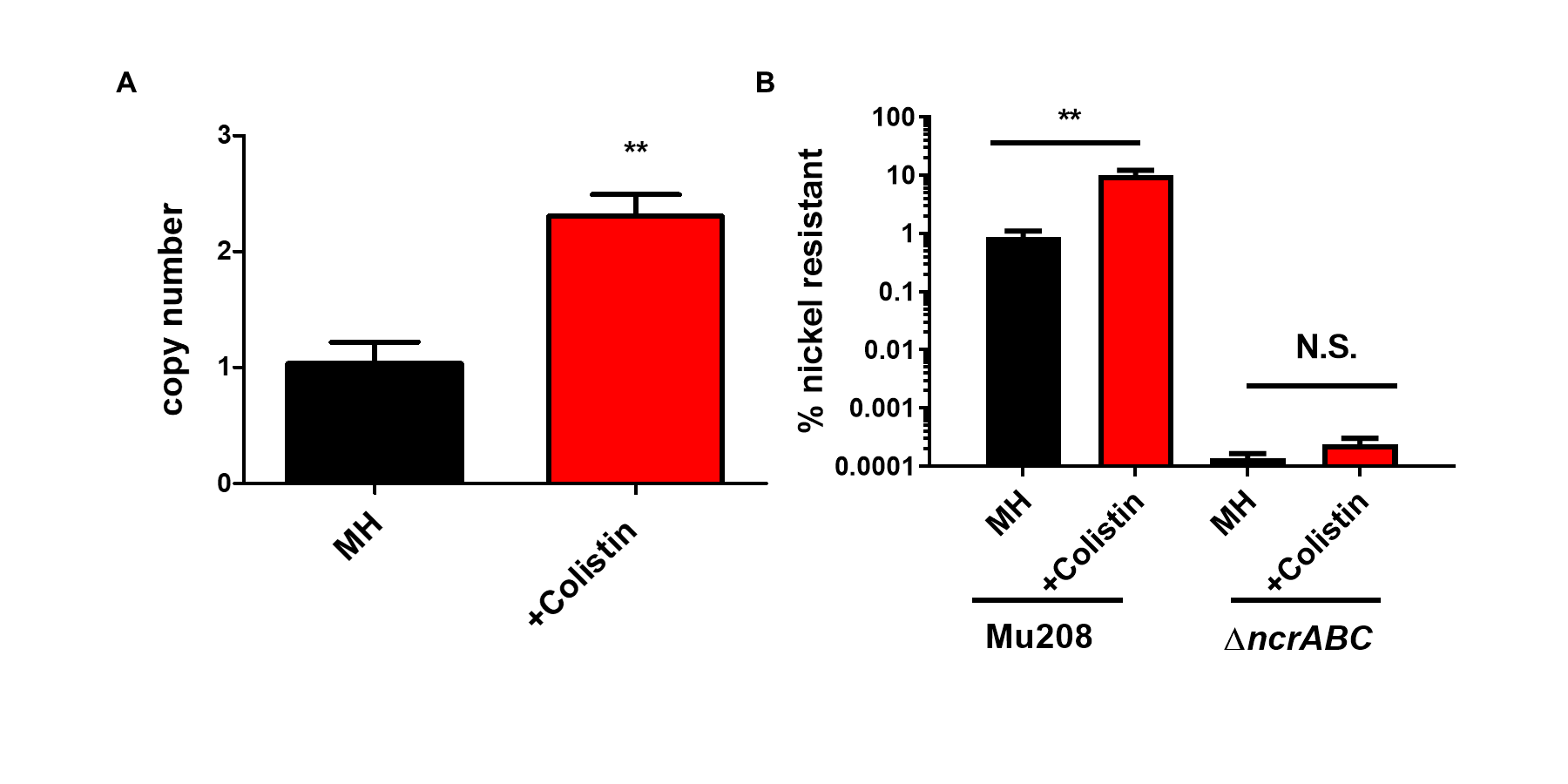

Supplement: FIG S6 [file mbio.02994-20-sf006.tif]

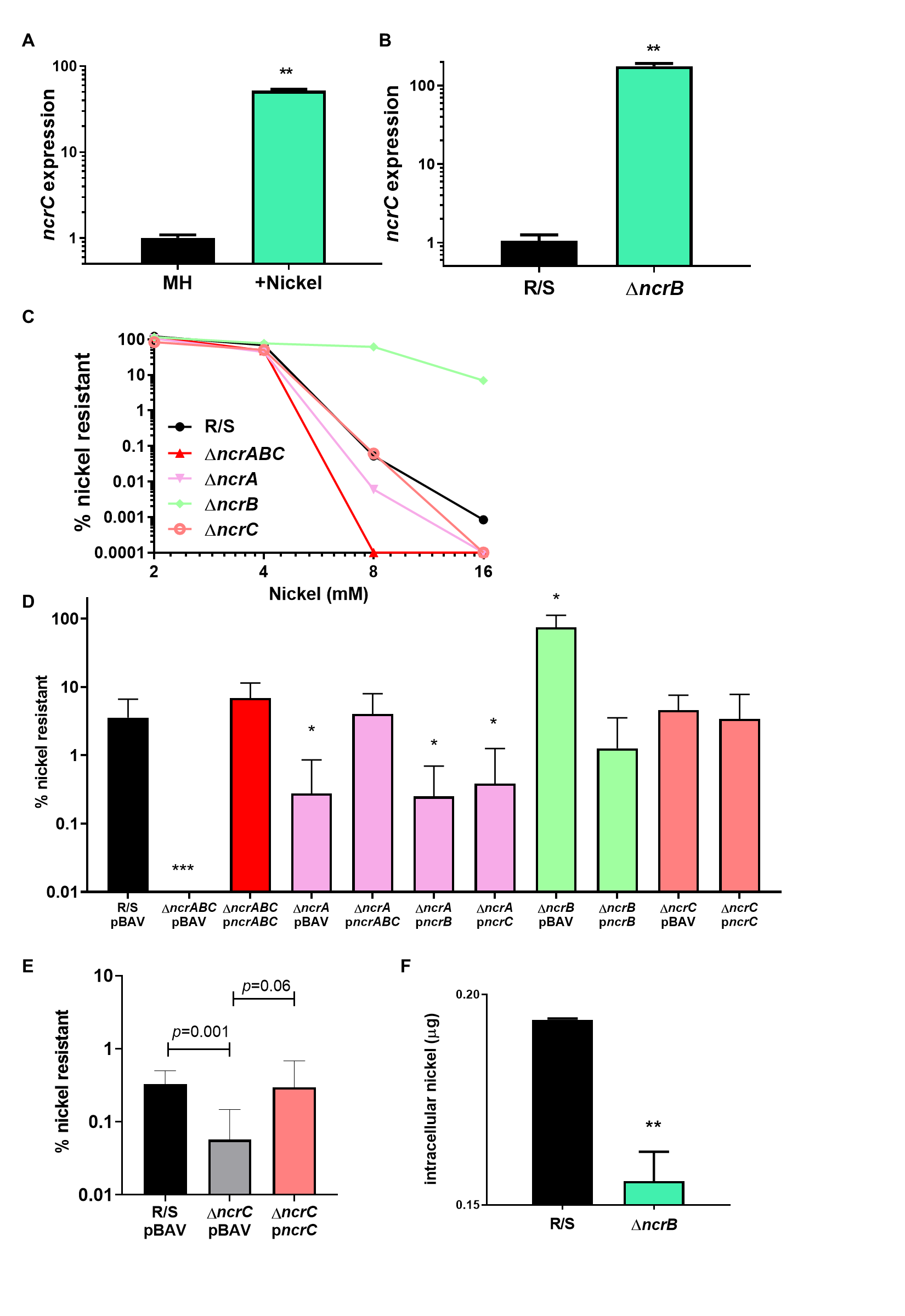

Supplement: FIG S7 [file mbio.02994-20-sf007.tif]

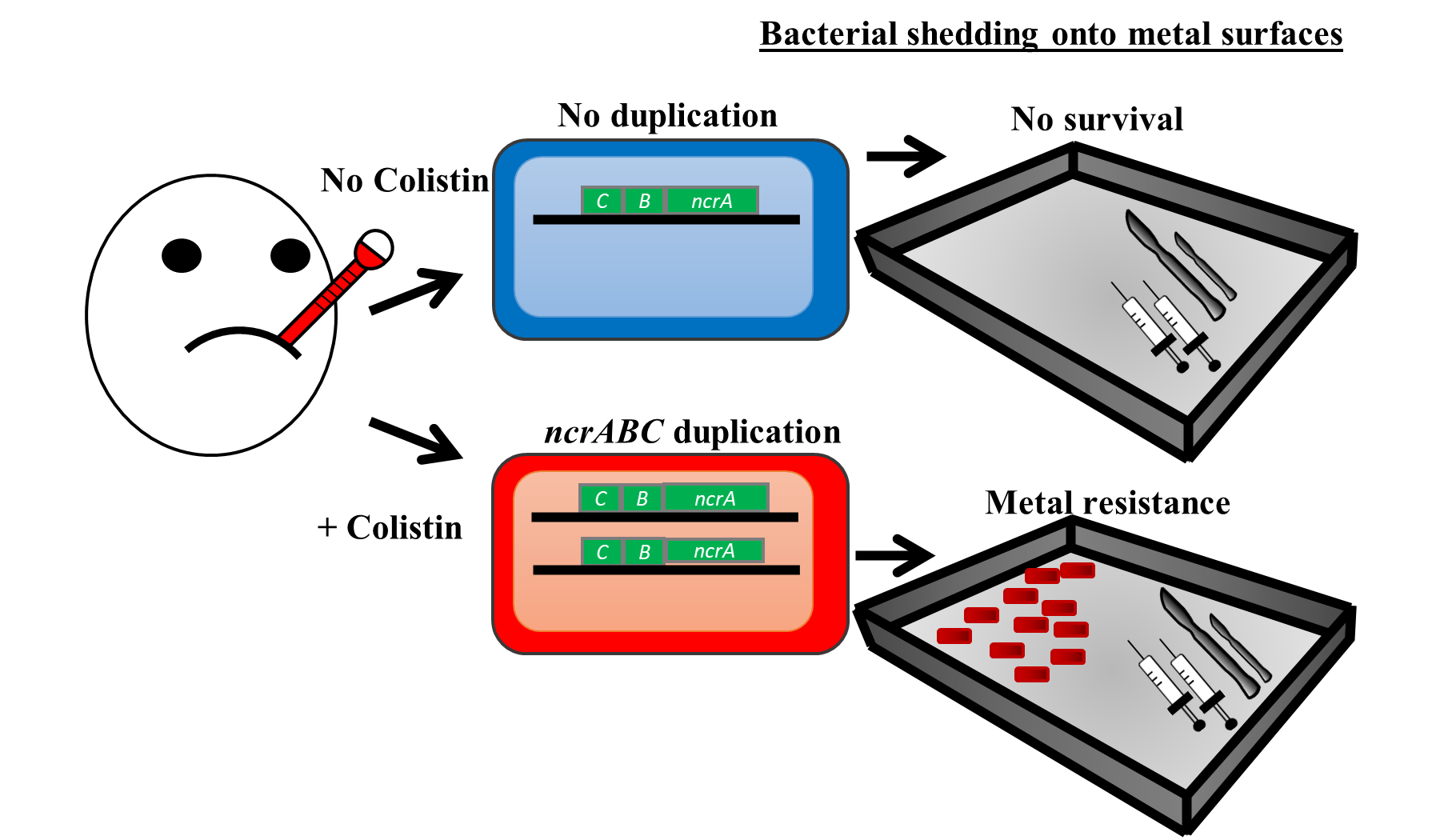

Supplement: FIG S8 [file mbio.02994-20-sf008.tif]
