# Supplementary material for: Antibiotic-Selected Gene Amplification Heightens Metal Resistance
Source: mBio. 2021 Jan 19;12(1):e02994-20. doi: 10.1128/mBio.02994-20 (PMC8545094; doi:10.1128/mBio.02994-20)
Supplement: TABLE S1 [file mbio.02994-20-st001.docx]

| **MIC to different antibiotics** | | |
| --- | --- | --- |
| **Antibiotic** | **R/S** | **Δ*ncrABC*** |
| **Pip/Tazo** | **>128** | **>128** |
| **Cefazolin** | **>64** | **>64** |
| **Cefoxitin** | **>64** | **>64** |
| **Ceftazidime** | **>64** | **>64** |
| **Ceftriaxone** | **>64** | **>64** |
| **Aztreonam** | **>64** | **>64** |
| **Ertapenem** | **<0.5** | **<0.5** |
| **Meropenem** | **<0.25** | **<0.25** |
| **Amikacin** | **<2** | **<2** |
| **Gentamicin** | **4** | **4** |
| **Tobramycin** | **8** | **8** |
| **Levofloxacin** | **>8** | **>8** |
| **Tetracycline** | **4** | **4** |
| **Tigecycline** | **2** | **2** |
| **Nitrofurantoin** | **64** | **64** |
| **SXT** | **<20** | **<20** |
| **Colistin** | **>256** | **>256** |
