# Supplementary material for: Antibiotic-Selected Gene Amplification Heightens Metal Resistance
Source: mBio. 2021 Jan 19;12(1):e02994-20. doi: 10.1128/mBio.02994-20 (PMC8545094; doi:10.1128/mBio.02994-20)
Supplement: TABLE S2 [file mbio.02994-20-st002.docx]

**Primer and nucleotide list**

| Primer or nucleotide | Sequence | Use |
| --- | --- | --- |
| 9kb RS F | AAT GAG TTA CCA GCT TTG TGG CTA CAT TTT AGC CTG AGG GAG ACA AAA CAG AGG TCG ACG GTA TCG ATA A | Primer used with 9kb RS R to create kanamycin casette for lambda red mutagenesis deletion of 9kb segment |
| 9kb RS R | TAA AAC CCA CCG GTT GGT GGG TTC AGG CGT GAG ATT CAT ACA GGA GAA AAG CAT AGC TGC AGG ATC GAT A |  |
| ncrABC RS F | TTT GAA TAT CCC CCT GGG GGG GAT GTG ATT TCA AAC CAA GAG ATT TCC TGG AGG TCG ACG GTA TCG ATA A | Primer used with ncrABC RS R to create kanamycin casette for lambda red mutagenesis deletion of 9kb segment |
| ncrABC RS R | TAA AAC CCA CCG GTT GGT GGG TTC AGG CGT GAG ATT CAT ACA GGA GAA AAG CAT AGC TGC AGG ATC GAT A |  |
| ncrA RS R | CACTTTTTATCGGCTTATCCTCTCCTGATACGTTGTCTTTTTTTGGGAAAGCATAGCTGCAGGATCGAT | Primer used with ncrABC RS F to create kanamycin casette for lambda red mutagenesis deletion of ncrA |
| ncrB RS F | ATGGCGTTGATACACATTATCTTATCCCTTCCACCTGGAGAGGATAAACCGAGGTCGATAA | Primer used with ncrB RS R to create kanamycin casette for lambda red mutagenesis deletion of ncrB |
| ncrB RS R | GCCACGCCTTGCTGTAAAAGAGTGGAAAAATCAGTCATGTTTCGCTTGCCGCATAGCTCGAGGATCGAT |  |
| ncrC RS F | CGCTTAGCGAATTTAAAGAGATAACTAAATATCTTTAAGGCAAGCGAAACGAGGTCGATAA | Primer used with ncrABC RS R to create kanamycin casette for lambda red mutagenesis deletion of ncrC |
| operon2 RS F | GAT TGC TAC ATA TTT TCA GTC TTT CTA ACC GTA CAT ATC GCT ATC TTT TTG AGG TCG ACG GTA TCG ATA A | Primer used with operon2 RS R to create kanamycin casette for lambda red mutagenesis deletion of 9kb segment |
| operon2 RS R | GAA GGT GTT ATC GCC GCT TCC CTG CGC AGC CCC TCC TGA CGA GAA GGG AAG CAT AGC TGC AGG ATC GAT A |  |
| pBAV-ncrABC F | GAT CGG ATC CCG GGT TTC CCG GTG ATG CCT TCC TGG GAT G | Primer used with pBAV ncrABC R to amplify ncrABC with XhoI and BamHI restriction sites for insertion into pBAV-gfp |
| pBAV-ncrABC R | GAT CCT CGA GTC AGC TCG TTA CGC CGA TCC AGC CGT GAT A |  |
| pBAV P_ncr_ F | GATCACTAGTTCCTGGGATGGCAACTCTGATACCTGGCCG | Primer to amplify ncr promoter with pBAV P_ncr_ R with homology to ncrB or ncrC |
| pBAV P_ncr_ R with homology to ncrB | GAACGGTCATCAGGAAATCTCTTGGTTTGAAATCA |  |
| pBAV P_ncr_ R with homology to ncrC | AAATCAGTCATGCAGGAAATCTCTTGGTTTGAAATC |  |
| pBAV ncrB F | AAGAGATTTCCTGATGACCGTTCATGCATCACACCC | Primer used with pBAV ncrB R to amplify ncrB for insertion into pBAV-gfp |
| pBAV ncrB R | GATCTCTAGATTAAAGATATTTAGTTATCTCTTTAAATTC |  |
| pBAV ncrC F | AGAGATTTCCTGCATGACTGATTTTTCCACTCTTTT | Primer used with pBAV ncrC R to amplify ncrC for insertion into pBAV-gfp |
| pBAV ncrC R | GATCTCTAGATCAGCTCGTTACGCCGATCCAGCCGTGATA |  |
| 9kb qPCR F | ATA GGG CTG ATG ACG CTT TC | Primer pair with 9kb qPCR R used to monitor gDNA of the 9kb region specifically in ncrAC operon |
| 9kb qPCR R | CCT ACT TTC CAG GCA ACT ATC C |  |
| rpoD qPCR F | CCG GAA GAC AAG ATC CGT AAA G | Primer pair with rpoD qPCR R to monitor gDNA of rpoD on the chromosome |
| rpoD qPCR R | CCT CGA TGA AAT CAC CCA GAT G |  |
| operon2 qPCR F | TCA AAG CCA AAG GCA AGG AT | Primer pair with operon2 qPCR R to monitor DNA of the 9kb region specifically in the operon 2 region |
| operon2 qPCR R | AAA AAT GCG TCC GAA TGT CC |  |

| 9kb | ttgtttttgcatagcagattccatttataattttacatcccccatgggggatattgtcgatgataaatgagcggctataaaagctcaattcctatccccggcagggggataaaaatatgatgggcttcagaaatataaaacccaccggttggtgggttcaggcgtgagattcatacaggagaaaatcagctcgttacgccgatccagccgtgatagcccatgtaaatgccgacaagtgcaatcagtacgcttgagaagtatggagcccgccgcgccagcgtatccaggccactccagcgtttggttgcctgacgaacgctgaaagccgccgctgcgcccactgaaaccagcgtgatcgctaaaccgatgctgaaacacagtaccagcgcggcaccgagcgtaaattccttcacctggatgcagagcagcaaaacggtaatggctgccgggcaaggaataaggccgccggtcaggccgaacaggattatttgtccggtggttacgctgcgattcgcaaaacgttttttgatgtcgttcgcatgcgctttttcgtgagcatcctgatattcgcgagaattaacatccagaccttccagcgaggaatgatcgtggtcgtgttcatgttcacggaattccacatcataatcatgcacatggccgcgatggcctaaagacaggcgaacatcaaagctgtgtggctccgggatcgccagggccgattccataaacccgtctttttcgacgaaatcaaatacctgtgaaaatgtccctggaccacggttggtcaccagagaaatgtcgcgagcttcccatttcttgccgctgagcgagcgtaaacgccagtgagggggttgtccttcttcgaaaatcgagagttcaacgctgccatggcccgtatcgatgatgcgggtttcgtcatgatcgtgatgaccgtggctgtgttcctcttcctgctccatcttccacagcttttcgccgcgccaggttcgccagaacatccatgccgccgtaccgagaatgatgacggcggaaatcagctgaaaccagggttcggctgattcagcagtgaatttctgactgatatacattccgccgaacgcaatgagccagacaacagacgtatgagaaattgtcgcggcaagccccagtaataccgcttgcttgaccgttccccggatggccacaataaaggcggccatcatcgttttggaatgccctggctccagtccgtgaagcgcaccgagcaaaatagcgctggggatgaacagccaggcattggccacgccttgctgtaaaagagtggaaaaatcagtcatgtttcgcttgccttaaagatatttagttatctctttaaattcgctaagcgtcttatcagactccgtgtggtcagcatgaagggcatcctccaggcagtgatccagatggtcatgaattaaggtacgcttcgcattcgttatcgccttttccacagcgtgcagttgctgggcgatatccagacatgctttctcatcttcgagcatctggatggtacttttcaggtgccctgcggcacgtttaagtcttttaataatgtcagggtgtgatgcatgaacggtcatggtttatcctctccaggtggaagggataagataatgtgtatcaacgccatggtcacactttttatcggcttatcctctcctgatacgttgtctttttttgggaaattacttctctttcttcggccaggacggatgttcatcgtcaatgacgaaatcatgctcatgggtcgattcgttgtttgtttccgggtctttgtggctgtgaacctgagcgtacacttcatgctcaggacgccagataagtacggcggtgaccagcgacaatgttgccacaacagtcagggcgatgaatgaagcctgtgtcccccatgccgcaccaacccatccggccagcggataagtcaccagccagcatgaatgtgacagtgcaaactgggcggcaaacagcgccggtctgtcttccgctgttgacgaacggcgcagcaggcgaccacccggtgtctgagaaagtgaatagcccacccccagcagtgcccagagcacaaccagcgtgatgtaatccttcaggaaaatgcctgaccccaggccaatgactaaaagcgccgtacctgcaagcatcggcatacggtcgctcatgttgtcgaggagtctggggagaaccagggccgataccatggaccccacgccgaagaaggccagcgcaatagcggtagaccgctgtgagaaaccaaagtccgcctgaaccagaacgacggtgttgacgataaccattgcactcgctgccgctacggccatgttgagtgccagcaggccgcgcagtctgggcgttttcaggaagatgcgcatacctcgtgtagttttgtcatagatgctgcgtttgaccgttaacgctttcatcttcggcagtgtcacggaaacaaccagagctgctgaagcaaggaacccgactgccgtaccggcaaacaggttgtggaaactcatgaccgtcagcagtgcagccgccagcatagggctgatgacgctttcaagatcgtaggccagacgtgacagagacagcgcccgggtatattcacgttcgtcaggaagaatatccgggatagttgcctggaaagtaggcgtaaacgccgccgaggcagcctgaagaatgaagatcaggatgtagatttcccatatctgggtgacaaaaggcagcgtgatagccacgagagcgcgaaccaggtccagtgttaccagcatggtgcgacgcgggaatttatcggcaaaagccgccgccacgggcgcaacaagaatataggcgctcattttgatagccagcgcggtacccagcactgccccggctctgtcacctgcgagatcgtaggccagtaaccctaacgcaacggtcgccagaccggtgccaataagggcgataacctgtgccataaacaggtggcgataggtacgattagaaagaatgttgagcatcaggaaatctcttggtttgaaatcacatcccccccagggggatattcaaagcatagagggacctgaaaaaaatgacaagctttatccgggtgaggaggatactgattcgtctgttatcttggatgttaaataaaaaagcttcaggtatcccagcgttatacctgaagcagcataaaaagtgtcgaaggtgttatcgccgcttccctgcgcagcccctcctgacgagaagggaattaactcagttgtcccagacaggttttcaggctcaaccgggtggcttcaatatcactatggataatggtgcaccagtcatcacggttagcccgaatgacctccagccagttattcagtgcctgattaaacgccatggttttctcgcgctgcggccagctcatctggttagtgctgtaaatgtcctgccctggctgcggcattagtacggtttgctgcgtcgacatccgggaagtcaccggccaggtatcagagttgccatcccaggaaggcatcaccgggaaacccgctccgcgcaaggccatatcccaggtggcatcaaacaggtatgttttgccctgtagctcaaaagccacagtgtcatgaaatccctgtgtttgcggcaaaattgccagaatatccgcaggtacaggcaaatcagcccagtcaaaccgcaccagctttcgctttgccgtaatgccaccatcggtcagcagtcgttcggcagcagcggctttatgacggcagtcgccaaagccctgctgaaacagactcatgctttcgccattccatgcaccgagctgatagggtatccgcctgaccagctcaaaaatggctcttcgcagttcagttgagccaggtttaagcccgagcgactgcaaaacccagcccggcgttatcgcctcttgttgattgcggcgggtgtttattttttgccatgtttcactgactttttcagccagagaggctgccagggtttcccccggcagtaatgttgtcatcagcaacgctcctgtggtggtgagaaaatgtctgcgcgtcaacatatcgggcgttacacctctacgttgcgacgaagcgccgacataaccagtgattcacagaccacttcttcatcctctgcccgctttacgccggggaagaccatctgacttaccggggacgaacgaagccggtgataacgggcaatcgcgccattcattcctcccatttgcttacgtctgaccagccgcagtccgtgctcccaggcgacttcatccatcgcgctcgccagaacagggtcatggttgaagatcaggacgccatcctctttcagcaggcgtgcagcgctgcgaaggaaagccgtgtaatcctgttcgaaataggccgctgcctgggagtagataatatcgacctcacctgcctgaacacctctgttttccagcgctcccagcccggctgctgtcacgtaaggatgctcagtcacgccttttggcagcgatgcatgccacggtgtgagattagtgaagtgcaggaatacgctttccccaagttgctccctgaactccccggaaagcccggaaatcgtcgcctgatggccggcacattcctcagcgcgaaaacccgcccaggcggcaccaatttccaggaaggtgactttcccgtttttgtggtgaatgtttttcgcatagtgggcgatgtcgaacccaatttctcgcgccaggcgaaatgaaccgccgctggatgtccaggggccatcaaagccaaaggcaaggatcgctttgagcatcgagcccgtttcctgatgcatcattttcaggaacggaatgactttacgccgtgaaggaaccgggacattcggacgcatttttttggcctgaaggtcgcgattaacgcccatgcagagcgtctgccagtgcaggggatcaacacgataattgagtgcagtgaaattacgcgcaatctctcgacgcgcctggatagagtctttatacatagcctctccgctaaatctgaagttgattaaagccgggcagcaaaaactgccggtaacagcgatcagatctggcggatgctgggaggacggtaaatgatgtccgtggccttaaccgggctacgaagatcggcaagcggcaaccatctgtctgttacaggagagagggaaatctctacagagggcggctgctcaggggtttcatggaaatggtctgtctgatgatggcctgcgtggccagactgagggatatccgaatcgtgagagtgatcctgcattgacatggtgacttctgcgtcatgcagattcatctcgtagcctgtcaggccgtttgtaccgtgcgacgcaaacgtcaccatcgacgacatcatcatgccaatggccatgatgacgatcatcagaacataacggggcgaacgtttaaacatcgggtacatcgctgattgctttcctcaatagacaatgacaacggtataaaaagattagcatccccccggggaggatgtaagggctggaatgtaaagtcttgtattggcgatatgaataaaaaggcccgcataatgcatcagcgaccggggcgaccagaatataagcaagcatcttcattgccagcgccgttcccgactccccctgccctgtcgcccgccagcaggcccagtgcaacggtagcaagaccggtaccgataagagcaattatgagcaccataaaaagatagcgatatgtacggttagaaagactgaaaatatgtagcaatctctaccatatgtattaatctctacactatgaaactacatatccttattctgacactgcccactgagcaggccacattacgtatgcgtgtctggcgggcattaaaaaataccggtgccgccatgctgcgcgacggcgtttatctgttgcctgaagcgcaacagagccatgagatttttaacgagatgagtcgtgaaatcagcggtgagggaggaacggcctttgtttttgacgcagagacttccgacgaagagaaaattcgcccgttattcgatcgctctcagcagtacctgatcctgatggagagccttcaggtttgtaaaaacgaccttaatgaggaaaccgcggtcagccaactgaagatggtaagaaaacttcgtcgcgagctggatcggattgtggcgattgatttctttcctggtgaagcgcaggcgcaggccatttttgccttgtcagaactggaagctggcattaacaggttcatctctcccggagagcctcatgccgtcagtggcttgctgacacggctgaagcctgaggattttcataacaggatctgggctacgcgtcgccggccatggattgacaggcttgccagcgcctggcttatacggcgttttatcgaccaggatgcgcagtttctctggcttaaggatggcaacgattgcccggaagaggcggtggggtttgattttgatggcgcgaccttcagccacatcgacaatcgtgtgacctttgaagtgctgatggtccgcttcgggctgacgggggatgccctgaatggtctggggatgcttgtgcattaccttgatgttggcggggttcagccccccgaagccgctggcgttgaaagtgtactggcaggattgcgtgaaagcattacggatgatgacacattactcacagcggcctgttcgctgtttgacggtctgttaacgacgtttgaaatgaggtccggccatgatgaacaaaacggagttgctgacgctggcagaggaaaacgttaagcccaagccggtccccctgaaggaggcgttctggttctggatgaagctgggttttatcagctttggcggtcctgccggacagattgcgatcatgcatcaggaactggtcgagaaccggcgctggatttccgagtcccgttttctccacgcactcaatttctgtatggtgctgcccggtccggaagctcagcaacttgccacctacattggctggctgatgcacaggaccttgggcggagtgatagcgggtttgttatttatccttccctcactttttattcttatcgcgttgtcgtggatctatatcgcctggggagacgttgccattattgccggtatcttttacggcattaaacccgccgtggcggcaatcgtgctgcaggcggcgcatcgcatcggctcccgtgcgctgaaacacggtgcccactgggccatagctgctgcggcttttgtggccgttttcgccctgaacgttccttttcccgtcatcgttatttcagcagcgataactggctttatcggcggacgcatcgcgccggaaaaatttcacagcggcagcgggcataacaaacaggagaaagccgccgttgatgctgcggttattgacgatcatacgcccgtaccggcgcatgccctgttcagctgggcgaaactgttaaggattgtggcagcgggtgcactgctctggcttattcccatgacggtgctgacgctggggctgggctgggcgcatccggtgacgcagatgggctggttctttaccaaggctgcgctccttaccttcggtggggcctacgccgtcctgccatacgtttatcagggtgctgtcgtcaattttggctggctgactgccgggcagatgatggacgggctggcgctgggagaaaccacgcccggcccgctgattatggtggtcacgtttgtcggatttgtgggcgggtatacccatgccgtttttggcgcggatatgctgtttgtcggcggcgccgttgccgcctgcatggtgacctggtttacctttctcccgtccttcattttcgtgctggcaggcgggccgtttatcgaaacgacccacaacaaggcgggtttcacggcacctttaaccgccatcacagcggccgtagtgggcgtcattgttaatctggggctgttttttatctggcataccgtatggccggaaggcgcaaagggagggatcgatatccctgccgccctgattgccgtcgccgctgcgtttgcgcttttcagattaaaatggaaggttacgcacgttattgccatggcggctctcgccgggctgatactccgcctgaccgggctgtcagctgtgtgaaaaggggcaggttatcctgcccctgatttatcagataacccgtttgatgttcaggtagtcgccggttgtatcaagctggatcgtgatggtctcagaaccacgatttcgccagaaccagccatgtttaccctcgaacgcggcctgtaaaacgccgtctccggatgtgatctgccgggctttaccatagccatggtagaagcctttcggggcgttgaccggatcgccgtgcgcatccacgtttaccggtccggaacttgtccactgataggtcacgcgggcatctttgcgcatttccagtttaacttcagcagcttcgttaggcttcagggtgatgagcatctgatgacgttgcgagccagccgatggtacttccggcgcgcgcgtggtcgccggaacgctcagctccgggtaggtgttctgcgttattgccggaggattatccgggatcggtcgctcattaactggcgcagccagctgtccttgctgaacagcgctggcctgattatcggcatcagcttcttcggcaagctgcgttttgatttctcccatctgcttaagccccagtaaacgacccacgccagtaggatcaatgccgtattccgaaggcatgacgacggtgatcagcagcgtcagcgccacgatggcggcgaggatagtggactgcatcagtttgatggcagaaggtaattctgcgcgtgttggtaaatcagtgttgtacattgttttgtctccctcaggctaaaatgtagccacaaagctggtaactcattaacagaaatcccgcgctcatcatcaccacattggcggtataagcgtgtcggaaaaagctcgccgtttgccgccagtagctcataacgatcaggatggcgctcagcgcgagcagctggccgatctcaacgccaatgttgaaggcaatcaggttgacaaacaggccatcgggagag | Full 9kb region that is duplicated under colistin treatment conditions |
| --- | --- | --- |
